# Advancing codon language modeling with synonymous codon constrained masking

**DOI:** 10.1093/nar/gkag166

**Published:** 2026-02-25

**Authors:** James Heuschkel, Laura Kingsley, Noah Pefaur, Andrew Nixon, Steven Cramer

**Affiliations:** Biotherapeutics Discovery Department, Boehringer Ingelheim Pharmaceutical Inc., Ridgefield, CT 06877, United States; Department of Biochemistry and Biophysics and Center for Biotechnology and Interdisciplinary Studies, Rensselaer Polytechnic Institute, Troy, NY 12180, United States; Biotherapeutics Discovery Department, Boehringer Ingelheim Pharmaceutical Inc., Ridgefield, CT 06877, United States; Biotherapeutics Discovery Department, Boehringer Ingelheim Pharmaceutical Inc., Ridgefield, CT 06877, United States; Biotherapeutics Discovery Department, Boehringer Ingelheim Pharmaceutical Inc., Ridgefield, CT 06877, United States; Department of Chemical and Biological Engineering and Center for Biotechnology and Interdisciplinary Studies, Rensselaer Polytechnic Institute, Troy, NY 12180, United States

## Abstract

Codon language models offer a promising framework for modeling protein-coding DNA sequences, yet current approaches often conflate codon usage with amino acid semantics, limiting their ability to capture DNA-level biology. We introduce SynCodonLM, a codon language model that enforces a biologically grounded constraint: masked codons are only predicted from synonymous options, guided by the known protein sequence. This design disentangles codon-level from protein-level semantics, enabling the model to learn nucleotide-specific patterns. The constraint is implemented by masking non-synonymous codons from the prediction space prior to softmax. Unlike existing models, which cluster codons by amino acid identity, SynCodonLM clusters by nucleotide properties, revealing structure aligned with DNA-level biology. Furthermore, SynCodonLM outperforms existing models on six of seven benchmarks sensitive to DNA-level features, including messenger RNA and protein expression. Our approach advances domain-specific representation learning and opens avenues for sequence design in synthetic biology, as well as deeper insights into diverse bioprocesses.

## Graphical Abstract

**Graphical Abstract:**
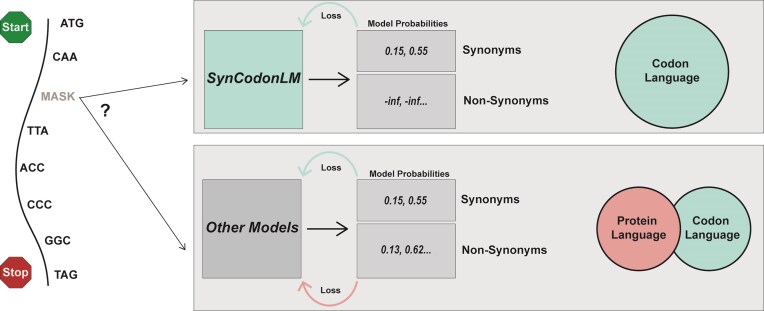

## Introduction

Codons are triplets of nucleotides that encode amino acids during the translation of messenger RNA (mRNA) into proteins. Although there are only 20 standard amino acids, the genetic code comprises 64 codons. This redundancy means that most amino acids are encoded by multiple synonymous codons. As a result, a single protein sequence can be represented by a vast number of different DNA sequences.

Codon optimization is the process of selecting a DNA or mRNA sequence that maximizes desirable traits, such as expression, without altering the amino acid sequence. Traditional methods often rely on heuristics, such as using the most frequent codons in the host organism or matching codon usage frequencies [1–11]. However, these approaches are limited in scope and do not capture the complex biological factors that influence translation efficiency, folding, and other aspects of gene expression.

Recent work has introduced codon language models to learn patterns in coding DNA sequences (CDS) using deep learning [12–17]. These models are inspired by protein language models, which have shown remarkable success in structure prediction and mutation modeling [18]. However, current codon language models suffer from a key limitation in how they are trained. During masked language modeling (MLM), the model is trained to predict a masked codon from the entire codon vocabulary, including codons that encode different amino acids. This means that when a codon for alanine is masked, the model is penalized not only for predicting incorrect synonymous codons, but also for predicting codons that encode a completely different amino acid, codons that are biologically invalid in that context. Penalties on non–synonymous predictions introduce semantic interference: the resulting gradients are conflated with amino–acid–level mismatches rather than explicitly aligned to codon–level patterns. As a result, parameter updates primarily suppress non–synonymous codons, diluting signal needed to discriminate among synonymous codons.

This training setup introduces a confounding factor. The model must first learn which amino acid belongs at a given position and then which codon is most appropriate for that amino acid. As a result, it becomes difficult to isolate patterns that are specific to codon usage and DNA-level features.

In parallel, techniques such as logit masking and targeted logit manipulation, where certain outputs are deliberately excluded or downweighted during prediction, have emerged as active research areas in the field of machine learning. These methods are particularly useful for enforcing known structural constraints and improving model interpretability [19–22], in this case, codon optimization, where the protein sequence is known *a priori*, and the objective is to optimize the corresponding DNA sequence. Logit masking has been used in generative codon language models to make sure codon output translates to the input protein sequence [23, 24]. However, these models differ fundamentally in their design: one operates on amino acid sequences and does not represent codons directly, while the other applies synonymous constraints only during inference, not during training. Our work extends these ideas to the training objective itself, demonstrating how biologically grounded logit constraints can be integrated into masked language modeling to guide representation learning. This approach aligns with broader efforts in ML to incorporate structured priors and task-specific constraints into foundational models and illustrates how training-time logit-space control can reshape learned representations in biologically meaningful ways.

Building on these insights, we introduce a new training strategy, synonymous codon-constrained masking. When a codon is masked, the model is restricted to predicting only codons that encode the same amino acid. For example, if the masked codon is “AGT,” which encodes serine, the model is only allowed to predict serine-encoding codons, “AGT” or “AGC.” This constraint is enforced by applying the softmax operation only over the subset of synonymous codons, rather than the full codon vocabulary. As a result, the model no longer needs to infer amino acid identity during training and can focus entirely on learning codon-level semantics.

Our model, trained with this strategy, outperforms existing codon language models on tasks that are sensitive to DNA sequence variation. Furthermore, analysis of the learned embedding space reveals that our model clusters codons based on nucleotide composition, while unconstrained models tend to group codons by amino acid properties, highlighting the conflation of codon usage with protein-level semantics in previous approaches. This makes our model particularly well-suited for applications in nucleotide-based therapeutics, such as mRNA vaccines, gene therapies, and oligonucleotide design. It also provides a powerful tool for codon optimization in biotherapeutics, where synonymous codon choices can significantly affect protein yield, folding, and other sequence-dependent properties, without altering the amino acid sequence. Beyond its biological utility, SynCodonLM illustrates how architectural and objective-level choices can disentangle latent factors in structured sequence modeling, offering insights applicable to other domains with symbolic or hierarchical vocabularies.

## Materials and methods

### Pre-training data

We curated a comprehensive dataset of CDS from the NCBI RefSeq database [25], encompassing nine major organismal groups: Archaea, Bacteria, Fungi, Invertebrate, Plants, Protozoa, Mammalian Vertebrates, Other Vertebrates, and Viruses. To ensure broad taxonomic coverage and minimize sampling bias, we included only one representative CDS dataset per species, explicitly excluding subspecies and cell line-specific entries. This strategy helped prevent overrepresentation of well-studied organisms and ensured a more balanced view of codon usage across the tree of life.

To prevent overlap of our train and test set, as well as reduce overfitting to highly repetitive sequences, we applied clustering per species at the nucleotide level using MMSeqs2 [26] with an identity threshold of 90% and a coverage threshold of 80%. This replicates the settings used to generate very popular biological pre-training datasets such as Uniref 90 [27], although we clustered at the DNA level rather than protein level to allow some extra synonymous variation even if proteins were more similar. This extra step seems to have been omitted from almost all other codon language models, besides CaLM [13], which clustered at the protein level at 40% identity using CD-HIT [28]. We believe this is a critical step in removing redundancy in CDS datasets and preventing models from overfitting to highly paralogous sequences.

Given the disproportionate abundance of CDS entries in certain groups (e.g. Bacteria and other vertebrates), we applied stratified sampling to balance the dataset, with an intentional emphasis on mammalian vertebrates to support downstream modeling objectives. Rather than stratifying against a total number of CDS within our dataset, we stratified to balance the total number of codons per group to be equal to, or no larger than our dataset’s mammalian sequences. We did this considering that, on average, mammalian CDS were much longer than some others, such as bacteria. This emphasis reflects the relevance of mammalian systems in biotherapeutics and mRNA-based therapeutics, where codon optimization plays a critical role.

In total, the final dataset comprised 43 526 014 CDS entries from 35 618 unique species. We randomly partitioned the dataset into 98% training (42 655 495 sequences) and 2% testing (870 519 sequences) subsets. This large-scale dataset provides unprecedented diversity for codon modeling and is approximately four times larger than that used in any previously published codon language model [12, 13, 15, 17]. All sequences were validated to ensure they contained only canonical nucleotides (A, C, G, T), were divisible by three, contained no internal stop codons, and had no 100% matches within the same species.

### Model architecture

SynCodonLM was trained as a masked language model, based on the DeBERTaV2 [29] architecture, tailored for learning representations of CDS. We utilized DeBERTaV2 due to its demonstrated performance improvements over traditional BERT models [29], which have been used in other codon language models [14, 17]. We selected DeBERTa for its disentangled attention mechanism and relative positional encoding, both of which are well suited for capturing the complex, context-dependent relationships inherent in CDS.

Unlike BERT, which adds absolute positional embeddings directly to token embeddings, DeBERTaV2 separates content and positional information within its attention mechanism. This disentangled attention allows the model to better generalize across varying sequence lengths and capture long-range dependencies. Additionally, DeBERTaV2 employs relative positional embeddings, which encode the relative distance between tokens rather than their absolute positions. This is particularly advantageous for CDS, where codon context often depends on local and distal relationships within the sequence, such as regulatory motifs or translational hotspots.

We initialized the model with a hidden size of 768, intermediate size of 3072, 12 hidden layers and 12 attention heads, and a max length of 1024 positions. This configuration is consistent with that of other codon language models [13–15]. The model was implemented using HuggingFace Transformers (version 4.48.3) and trained using PyTorch Lightning (version 2.4.0) and PyTorch (version 2.7.1 + cu126) with mixed precision to optimize memory usage and training speed.

A custom tokenizer was built to include all 64 codons and 5 special tokens. During tokenization, each CDS is prepended with a [CLS] token and terminated by a [SEP] token to denote sequence end. We incorporated token-type embeddings into our input representation to give context relative to species for the model to make more informed decisions. While prior works such as CodonTransformer did this per species, our dataset comprises >35 000 species; therefore, the size of the token type ID embedding would become extremely large. Meanwhile, their dataset only trained on roughly 150 species [15]. Because of this, we limited token type IDs to a max of 501. This design balances biological specificity with computational scalability, allowing the model to incorporate species-level codon bias without inflating the token type embedding matrix.

As taxonomic groups in our dataset did not have relative sequence to species ratios, we decided to break down the allocation of those 501 token type IDs by the number of CDS per taxon. To group species together within each taxon, we calculated the RSCU for each species and then performed k-means clustering to assign token type ID across species with similar relative codon usage. This clustering strategy ensures that species with similar translational mechanisms are embedded similarly, improving the biological relevance of the learned representations. A final token type ID was reserved as “unknown” to allow model usage without species-specific embeddings.

### Model training

The model was trained using an AdamW optimizer with a learning rate warmup from 0 to 2 × 10^−4^ over the first 10% of steps, followed by a cosine decay back to 0. A dropout rate of 0.1 and weight decay of 0.01 were applied to reduce overfitting and improve generalization. These hyperparameters were selected based on prior work in transformer-based biological sequence modeling.

To increase biological specificity to CDS, we constructed a custom masking matrix of shape [*V,V*], where *V* is the vocabulary size, mapping each tokenized codon to only synonymous codons. During training, for each masked token, the corresponding row from the matrix is added to the models’ logits before softmax and computing the loss. This ensured that the model could only predict codons encoding the same amino acid as the masked token.

Equation 1:


\begin{eqnarray*}
\left[ {{{z}_{i,v}}} \right]\ = \ \left\{ {\begin{array}{@{}*{1}{c}@{}} {{{f}_\theta }{{{\left( {{{x}_M}} \right)}}_i}\left[ \nu \right]}\\ { - inf} \end{array}} \right.\begin{array}{@{}*{1}{c}@{}} {if\ \textit{synonymous}}\\ {if\ not\ \textit{synonymous}} \end{array}.
\end{eqnarray*}

Here, $[ {{{z}_{i,v}}} ]$ denotes the synonym constrained logit at position $i$ at vocab dimension $v$. The term ${{f}_\theta }{{( {{{x}_M}} )}_i}[ \nu ]$ refers to the original model logit produced by the language model ${{f}_\theta }$ for the masked input $( {{{x}_M}} )$ sequence at position $i$ for token $\nu $. For each masked token, the model’s original output is retained only for codons that are synonymous with the true amino acid, while logits for non-synonymous codons are set to negative infinity, effectively removing them from the prediction space.

Equation 2:


\begin{eqnarray*}
{{\mathcal{L}}_{MLM}} = - \frac{1}{{\left| M \right|}}\mathop \sum \limits_{i\in M} \log p\left( {{{x}_i}{\mathrm{|}}{{x}_M}} \right).
\end{eqnarray*}

In this formulation, ${{\mathcal{L}}_{MLM}}$ is the masked language modeling loss, computed as the average negative log likelihood over a set of masked positions $M$. For each masked position $i\in M$, the model predicts the original token ${{x}_i}$ given the masked input ${{x}_M}$. The loss is normalized by the number of masked positions $| M |$ to account for variability in sequence length and masking density.

This synonym-constrained training strategy restricts the model’s language space to biologically valid codon substitutions and prevents penalization for predicting codons that encode different amino acids. By aligning the training objective with biological constraints, the model learns codon-level variation independent of protein semantics. Accuracy was monitored over masked positions throughout training to ensure convergence.

The model was trained with an effective batch size of 1560, distributed across 12 NVIDIA L40S GPUs. We utilized 16-bit mixed precision training in PyTorch Lightning to reduce memory usage and accelerate computation. Training was conducted over ~8 days, spanning 3 epochs and 82 028 total steps. With an average of 407 codons per sequence, the model processed ~100.1 billion tokens, making it one of the largest codon language model training efforts to date.

### Input embedding evaluation

We evaluated the static input embeddings learned by SynCodonLM by passing all 64 codon tokens through the model and extracting their corresponding embeddings from the input layer. These embeddings reflect the model’s initial representation of codons prior to any contextual transformation and are useful for assessing how codon-level features are encoded.

To visualize the structure of the embedding space, we applied t-distributed stochastic neighbor embedding (t-SNE) using scikit-learn [30] (version 1.5.1) and NumPy [31] (version 1.26.4) to reduce the 768-dimensional embeddings to two dimensions. t-SNE is a non-linear dimensionality reduction technique that preserves local structure and is well-suited for visualizing high-dimensional biological data. We used default t-SNE settings for all visualizations (perplexity = 30).

The same procedure was applied to generate Supplementary Fig. S4a–e, which shows other codon language models’ embedding space. This comparative analysis highlights differences in how models encode codon relationships, particularly with respect to nucleotide-level versus amino acid-level clustering.

Additionally, we performed PCA [scikit-learn default settings (version 1.5.1)] on the learned token type embeddings corresponding to species clusters. These embeddings were derived from the RSCU-based k-means clustering described earlier and reflect species-level codon usage patterns. Visualizing these embeddings allowed us to assess whether the model captured biologically meaningful relationships between species based on codon bias.

### Evaluation datasets

We curated seven evaluation datasets in which different CDS encode the same protein but result in measurable differences in biological properties. These datasets were specifically chosen to assess the model’s ability to capture codon-level variation independent of protein sequence, aligning with SynCodonLM’s design objective. By holding the amino acid sequence constant, these tasks isolate the functional impact of synonymous codon changes.

The set includes “mRNA Toxicity 1” and “mRNA Toxicity 2,” both derived from a study [32] that observed cell death in *Escherichia coli* when codon-optimizing GFP. These datasets involve libraries of GFP codon variants expressed under similar conditions, but “mRNA Toxicity 2” introduces a 28-codon N-terminal tag designed to reduce mRNA secondary structure and has a much smaller sample size (*n* = 97) compared to “mRNA Toxicity 1” (*n* = 185), which likely contributes to greater variability and lower average R² values. We also included “GFP Expression,” which involves a study [33] that measured protein abundance changes in *E. coli* resulting from synonymous variation in the first eight codons of GFP and contains 219 sequences. Similarly, the “mRFP Expression” dataset [34] consists of measurements of red fluorescent protein expression from a large library of synonymous variants expressed in *E. coli* (*n* = 1459), where the authors randomly mutated the entire mRFP CDS.

In addition, we incorporated three datasets from a study [10] in *Saccharomyces cerevisiae*: “GFP mRNA Abundance 1,” “GFP mRNA Abundance 2,” and “TDH3 mRNA Abundance.” These measure transcript levels when shuffling different regions of GFP or the endogenous TDH3 gene, with sample sizes of 1124, 2432, and 523, respectively. Notably, “GFP mRNA Abundance 1” exhibits significantly lower mean R² compared to the other two, likely due to a highly skewed distribution toward the mean, which required Yeo-Johnson scaling, and because synonymous mutations occur in a region of GFP with minimal impact on mRNA stability compared to “GFP mRNA Abundance 2.” Together, these datasets span both prokaryotic and eukaryotic systems and reflect diverse biological outcomes, including expression level, toxicity, and mRNA stability.

Evaluation dataset attributes are summarized in Supplementary Table S1. All datasets were min-max scaled to preserve their original variance and comparability across models, except for one dataset with extreme skew toward a central mean and distinct outliers, which was scaled using the Yeo-Johnson transformation implemented in scikit-learn [30] (version 1.5.1). This preprocessing ensured that model comparisons were not biased by differences in scale or distribution.

### Fine tuning and benchmarking

To benchmark SynCodonLM against existing codon language models, we performed fine-tuning on our evaluation datasets using five widely adopted implementations: CaLM [13], CodonBERT [14], CodonTransformer [15], cdsBERT [12], and Mistral-Codon [16]. We also attempted to include EnCodon [35], but were unable to extract embeddings due to unresolved bugs in its codebase, which were documented in its GitHub issue tracker by other users as well.

All models had an embedding dimension of 768, except for cdsBERT, which uses a 1024-dimensional embedding space. To ensure comparability across models, we projected cdsBERT’s embeddings to 768 dimensions using Sparse Random Projection (SRP) [36], implemented in scikit-learn (version 1.7.1). SRP was chosen over principal component analysis (PCA) because several evaluation datasets contained fewer samples than dimensions, violating PCA’s requirements. SRP is a randomized linear projection method that preserves pairwise distances in high-dimensional spaces. This dimensionality reduction allowed for consistent downstream processing across all models.

Given the relatively small size of the evaluation datasets, we froze all base layers of each model and applied mean pooling across the sequence dimension to obtain a single embedding per CDS. A linear regression head was trained on these pooled embeddings using mean squared error loss, with both L1 and L2 regularization to prevent overfitting. We used a batch size of 16 and trained for 100 epochs per model-dataset pair.

To ensure robust performance estimates, we performed five-fold cross-validation and repeated the process across 20 to 50 random seeds, depending on dataset size. This repetition enabled the generation of paired samples for statistical testing, allowing us to compare SynCodonLM’s performance against other models using paired t-tests. Paired t-tests were two-sided with significance level α = 0.05. Mean performance metrics across folds and seeds were reported for each model and dataset.

### Effect of synonymous mutation on model performance

To quantify convolution between codon and protein-level semantics, we conducted a study similar to that described in the CaLM paper [13]. We obtained protein abundance data for *C. griseus* from the Protein Abundance Database (PaxDb) [37] and retrieved corresponding CDS from NCBI [25] to match the protein identifier. For each CDS, we generated synonymous variants at mutation levels ranging from 10% to 100% in 10% increments, ensuring that codons were not replaced by themselves during subsampling.

To assess performance degradation, we trained models using the same strategy as for the seven benchmarking datasets, but with a batch size of 64 to accommodate the larger dataset (*n* = 5528). We performed five-fold cross-validation over 10 random seeds and measured the performance of a model trained on non-mutated CDS when predicting mutated CDS at each level of synonymous mutation.

### Ablation models architecture

All ablation models were trained with a hidden size of 320, intermediate size of 1280, 6 hidden layers, and 5 attention heads. This yielded a model with a total of 9 million params in comparison to the full-size model of 102 million params. We reduced hidden size, intermediate size, and number of layers to maintain a proportional depth-to-width ratio with the full base model. We trained for one full epoch with an effective batch size of 800, roughly 1/3rd the size of the full model pretraining, and we decreased the max learning rate to 1e^−4^ from 2e^−4^ to compensate for the smaller batch size. Each model was trained for one epoch using the same full dataset from the full pretraining. All other training procedures were maintained from the full model pretraining.

### Ablation of non-synonymous mask

To evaluate the change in learned embeddings and performance given our model architecture, we performed various ablation studies. We trained a variant without synonym-constrained masking and compared it to a model of the same architecture trained with the synonym-constrained mask.

### Ablation of species group token type Id

Next, we trained a model with nine token type IDs corresponding to the NCBI base-level identifiers (Archaea, Bacteria, etc.). Additionally, we trained a model without the use of token type ID at all. Both ablations were to visualize the effect of our species token-type ID. These were compared to a model of the same architecture trained with the species token type ID as in the full model.

### Necessity of full pre-training with non-synonymous mask

We performed fine-tuning of our ablation model trained without the non-synonymous mask, with the non-synonymous mask. We fine-tuned with a similar strategy as pre-training, utilizing 5% of the overall dataset used for pre-training. Additionally, we used a max learning rate of 5e^−4^ for a total of 1 epoch (rather than 1e^−4^). It is difficult to clearly define the difference between “pre-training” and “fine-tuning”; however, this fine-tuning procedure required ~5% of the compute used for full pre-training.

### AI usage

We utilized Microsoft Copilot to assist with drafting and refining portions of the manuscript text, including figure legends, supplemental descriptions, and select sections of the Methods. The model was used to improve clarity, consistency, and scientific tone and to streamline the writing process. All content generated with AI assistance was thoroughly reviewed, edited, and approved by the authors to ensure accuracy and integrity.

## Results

### Motivation for synonymous codon-constrained training

Large language models (LLMs) developed for proteins have significantly influenced the design of codon language models. While standard masked language modeling (MLM) approach could be used, predicting masked tokens from the full codon vocabulary, we hypothesized that allowing non-synonymous codons in the prediction space introduces a confounding factor.

This conflation of languages was exemplified in the CaLM model [13], which maintained high performance on protein property prediction even when the input CDS was recoded such that every codon was replaced by a synonymous alternative. If a model relies exclusively on codon–level signals, performance should significantly deteriorate under a 100% synonymous recoding; the persistence of accuracy therefore indicates dependence on amino–acid–level information in addition to codon features. Notably, CaLM [13] was proposed primarily for protein-level tasks, unlike other codon language models, which do not make this distinction. Such findings reinforced our motivation to constrain the model’s output space to only synonymous codons during training, thereby encouraging the model to learn codon usage patterns independently of protein-level semantics.

### SynCodonLM architecture and species-aware training

**Figure 1.**
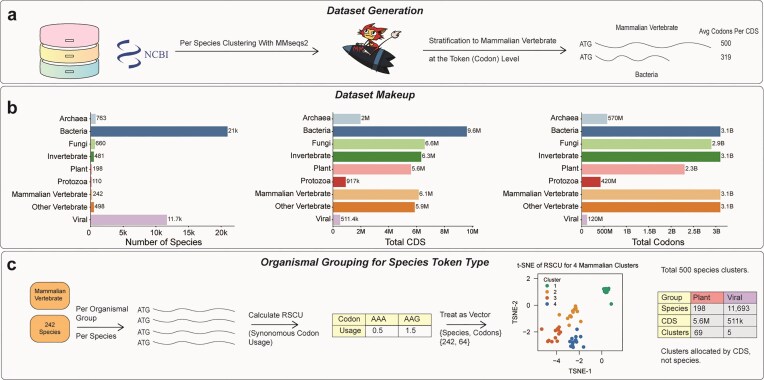
Dataset Curation and Species Clustering. (**a**) Using NCBI RefSeq database, we cycled through all 9 of its major organismal groupings, along with all species (while excluding cell lines and subspecies), to gather CDS. CDS were clustered per species using MMseqs2 at the nucleotide level with a 90% identity threshold. The dataset was stratified down so that no organismal group had more codons in the dataset than that of mammalian vertebrates. (**b**) Dataset makeup by species, total number of CDS, and total number of codons per NCBI major organismal group. (**c**) RSCU was calculated per species. RSCU was treated as a vector across all species in an organismal group and k-means clustering was used to cluster species with similar codon usage. These clusters are visualized using t-SNE, where species with similar codon usage patterns (colored by cluster) appear closer together in the embedding space.

Our model was trained on the largest dataset used to date for any codon language model, comprising over 43 million coding sequences (CDS) sourced from NCBI RefSeq [25] (Fig. 1a). These sequences span >35 000 unique species, offering unprecedented diversity and coverage across the tree of life. Unlike all other codon language models, besides CaLM [13], we clustered our pretraining dataset to remove highly redundant sequences, which has very likely led to overfitting in other pre-existing works. We clustered sequences per species, using MMSeqs2 [26] at the 90% nucleotide similarity level. We then stratified our dataset away from over-abundant taxa, such as bacteria, so that each organismal group had no more codons (tokens) than that of mammalian vertebrates. Final dataset statistics are visualized in Fig. 1b.

Inspired by the CodonTransformer model [15], we introduced learnable species-level differentiation into SynCodonLM by incorporating token type embeddings. To define each token type, we captured codon usage patterns across species and computed relative synonymous codon usage (RSCU) [6] values for each (Fig. 1c). These RSCU values were treated as vectors and clustered using k-means to assign species with the most similar RSCU to the same token type ID. An example of four species clusters and their t-SNE values is seen in Fig. 1c, along with the rest of this clustering setup. These species clusters (Token Type ID) seem to show some level of clustering by their taxonomic group, as shown in Supplementary Fig. S1.

**Figure 2.**
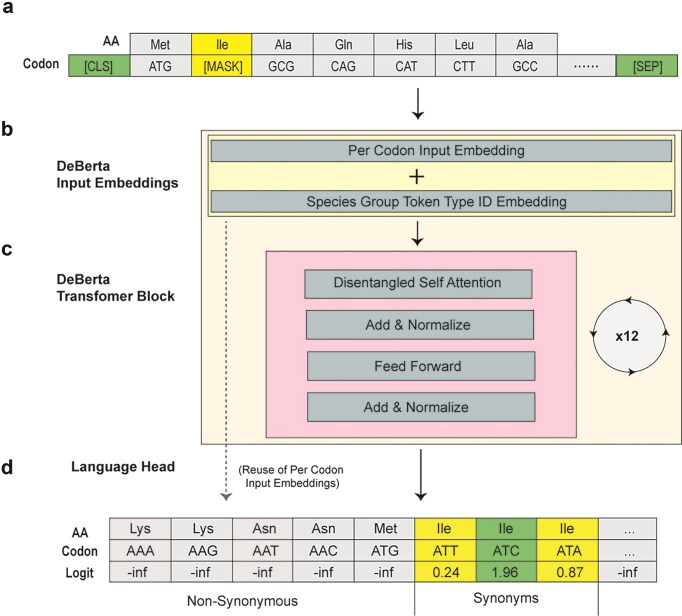
Constrained Decoding of Model Output During Training. (**a**) SynCodonLM masks roughly 15% of input codons. (**b**) Token inputs receive their static, respective embeddings, which are each added to a static embedding for the species group, token type ID. (**c**) Input embeddings are fed into the DeBerta transformer block, where they have disentangled self-attention applied utilizing relative positional embeddings. Our model has 12 layers of this transformer block. (**d**) The language head computes per-token probabilities by projecting the final hidden states onto the shared embedding matrix used for input representations. Model output logits are masked with -inf for positions that correspond with non-synonymous codons.

During training, 15% of codons were randomly masked (Fig. 2a), and the model was tasked with predicting the masked codon using a DeBERTa-style transformer architecture (Fig. 2b and c). DeBERTa (Decoding-enhanced BERT with disentangled attention) represents a state-of-the-art advancement in BERT-based models, achieving top performance across a wide range of natural language understanding (NLU) benchmarks [29]. Its disentangled attention mechanism and enhanced position encoding allow for more expressive and efficient representation learning, making it particularly well-suited for capturing subtle patterns in biological sequences. Training convergence was stable across epochs, as shown in Supplementary Fig. S2.

Crucially, we applied a synonym-aware logit masking strategy at the output layer (Fig. 2d), where logits corresponding to non-synonymous codons were set to negative infinity. This biologically grounded constraint ensures that the model learns codon-level variation independent of amino acid identity.

The MLM accuracy on these constrained predictions reaches nearly 59%, as seen in Supplementary Fig. S3. Prior work has used a bi-directional RNN architecture to predict next codons in a dataset of CHO CDS [38], which utilized constrained decoding, and its accuracy was relatively lower at 53%.

### SynCodonLM captures DNA-level features, not protein-level properties

To validate that our synonym-constrained training strategy produces a model that focuses on coding DNA characteristics rather than protein-level semantics, we analyzed the learned input embeddings of all codon tokens in the vocabulary.

Previous codon language models [12–16] have shown that codon embeddings tend to cluster based on amino acid properties. In Supplementary Fig. S4a–e, we performed a t-SNE on the input embeddings of five other codon language models, which consistently reveal clustering by amino acid identity and physiochemical property. These models were trained without synonymous constraints, and their clustering by amino acid identity indicates an entanglement of codon and protein semantics in the learned representations, likely arising from the model having to infer the correct amino acid before selecting a codon. In contrast, our model, SynCodonLM, was trained to focus exclusively on synonymous codon variation, allowing it to capture codon-level variation independent of protein semantics, focusing on sequence features relevant to both DNA and mRNA.

**Figure 3.**
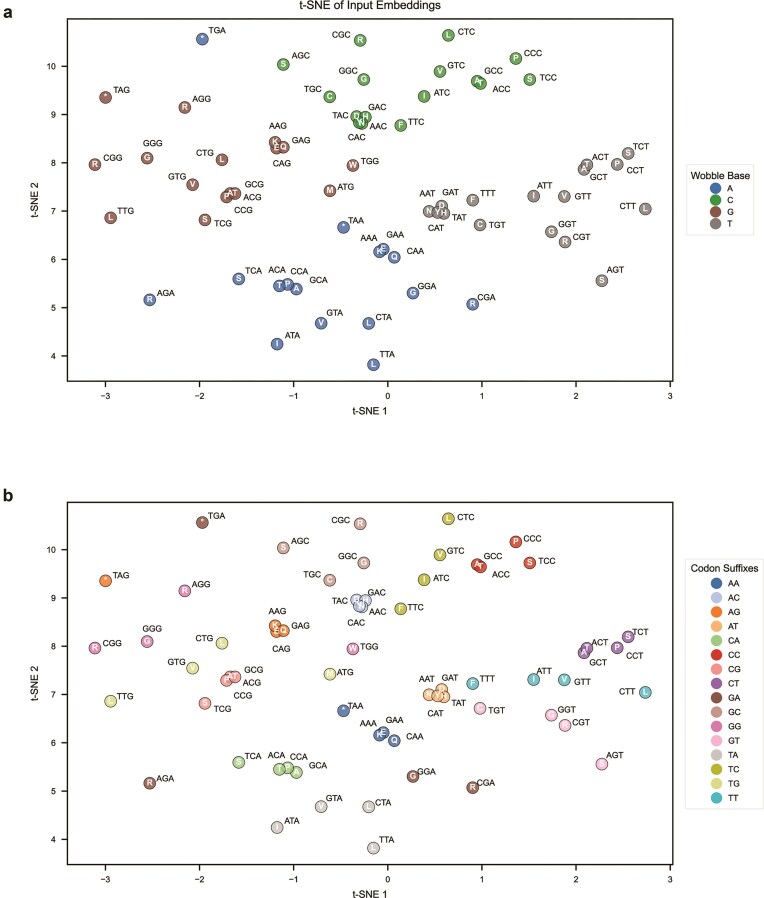
SynCodonLM Learned DNA-Specific Features Within Codons. (**a**) t-SNE projection of input embeddings for all codons reveals that codons cluster according to their third (wobble) base. Each point is colored by its wobble nucleotide (A, C, G, or T), highlighting the influence of the wobble position on the learned embedding space. Separation by wobble base is statistically significant (PERMANOVA, pseudo-F = 3.339, *P* < .001). (**b**) When colored by codon suffix dinucleotide (positions 2 and 3), the same t-SNE projection shows that codons with similar suffixes tend to group together, suggesting that the model captures dinucleotide-level patterns in codon representation. Separation by suffix dinucleotide is statistically significant (PERMANOVA, pseudo-F = 2.002, *P* < .001).

When visualizing the input embeddings of SynCodonLM using t-SNE, we observed that codons clustered based on nucleotide-level features. In Fig. 3a, codons are grouped by their wobble base, which is the third nucleotide in the codon. This clustering may reflect biologically relevant structure, as the wobble position plays a critical role in tRNA recognition and codon degeneracy [39], has been shown to influence translation speed [8, 9, 40], and mRNA stability [41].

In Fig. 3b, codons are grouped by their dinucleotide suffix, which consists of the last two nucleotides of the codon. Codons with the same suffix cluster together regardless of the amino acid they encode. This clustering may also reveal further biologically relevant structure, as there have been findings that suffix dinucleotides can induce unique structural changes in mRNA [42], and that these structural features can significantly alter translation dynamics [43, 44].

Both clustering patterns were statistically significant, as confirmed by PERMANOVA tests with a *P*-value <.001. Additionally, codons that do not have any synonymous alternatives, such as “ATG” for methionine and “TGG” for tryptophan, appear near the center of the t-SNE plots in Fig. 3a and b. This suggests that the model did not learn strong embedding features for these codons, which is expected given their lack of synonymous variation. Most importantly, there is no evidence of clustering by amino acid identity, which supports the conclusion that SynCodonLM learns nucleotide-driven representations rather than protein-driven ones. To confirm the robustness of these clustering patterns, we performed a sensitivity analysis on the t-SNE perplexity parameter. Grouping by wobble base remained consistent across all tested settings (Supplementary Fig. S5), supporting the stability of the observed structure.

Finally, we note an important architectural detail relevant to how these embeddings are used during prediction. Although input embeddings are a static lookup table, they play a critical role in language models. Nearly all transformer-based architectures, including BERT [45], GPT [46], and DeBERTa [29], employ weight tying in their language heads [47, 48]. This means the same embedding matrix used for input representation is reused to project final contextual embeddings into the vocabulary space. The projection is implemented as a dot product between the final hidden state and each token embedding, producing logits for probability estimation. Because the language head directly depends on these input embeddings for output scoring, the organization and grouping of these embeddings is especially important. Any biologically meaningful structure learned during training, such as clustering by wobble base or dinucleotide suffix, can influence prediction behavior and interpretability. This design ensures consistency between input and output spaces, improves parameter efficiency, and reinforces the importance of embedding geometry in shaping modeloutputs.

### SynCodonLM embeddings outperform existing models on codon-sensitive tasks

To assess the utility of the embeddings produced by SynCodonLM, we evaluated model performance across multiple datasets in which codon usage varies while the encoded protein sequence remains constant. These datasets were specifically chosen to reflect biologically meaningful differences in mRNA expression, protein expression, and cell toxicity driven by mRNA that arise from synonymous codon variation (see the “Materials and methods” section for details). This contrasts with prior studies, where evaluation datasets often include differences in both coding DNA and protein sequence, making it difficult to disentangle codon-level effects from protein-level semantics.

This distinction is critical. Models such as CaLM [13], cdsBERT [12], CodonBERT [14], CodonTransformer [15], and Mistral Codon [16] are trained using unconstrained objectives that allow them to learn a mixture of codon and protein language. As a result, their embeddings may reflect amino acid properties rather than DNA-level features. In contrast, SynCodonLM was trained using a synonym-constrained masking strategy, ensuring that its embeddings capture codon-specific signals independent of protein identity.

**Figure 4.**
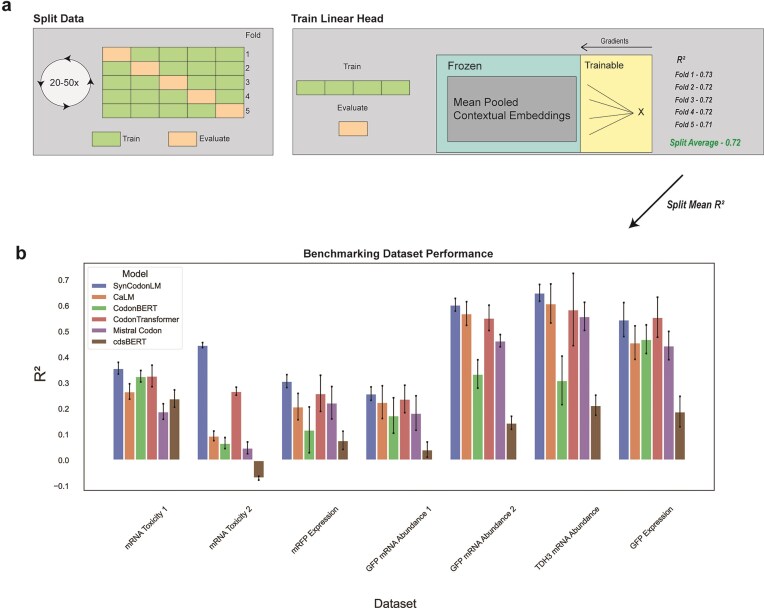
SynCodonLM Outperforms Other Models. (**a**) We performed five-fold cross validation on each dataset, training a linear head on mean pooled embeddings from each model for each of the five folds. For each set of five folds, we aggregated the split’s evaluation metrics. We re-split data 20–80 times to get 20–50 average R^2^ for each split, per model, dataset. (**b**) SynCodonLM outperforms all five other codon language models in six out of seven datasets (*P* < .05, paired t-test). SynCodonLM performs better than four out of five other codon language models in another dataset (*P* < .05, paired t-test).

As shown in Fig. 4a, we performed five-fold cross-validation on each dataset, repeating the process across 20–50 random seeds. For each seed, mean-pooled embeddings served as input to a linear regression head trained to estimate the fitness metric for each dataset. In our setup, we only train the linear head, which yields an equal and simple assessment of each model’s contextual embeddings out of the box. Average R² scores were then computed across folds, and these averages were used to calculate the mean performance per model and dataset, and to perform paired t-tests comparing SynCodonLM against other models.

The results, shown in Fig. 4b, demonstrate that SynCodonLM achieved the highest performance in six out of seven datasets, significantly outperforming all five other evaluated codon language models (*P* < .05, paired t-test). In the remaining dataset, SynCodonLM ranked second, outperforming four out of five models (*P* < .05, paired t-test). Model parameter comparisons are provided in Supplementary Fig. S6, demonstrating that SynCodonLM is similar in parameter count to other state-of-the-art models.

These findings support the assertion that SynCodonLM learns biologically meaningful DNA-level representations, making it better suited for tasks where synonymous codon variation drives functional outcomes. These results demonstrate that constraint–aware training can yield representations that capture codon–level signals and a distinct latent space, providing a framework for structured sequence modeling in other domains.

### Ablation studies to validate effectiveness of synonym-constrained masking

**Figure 5.**
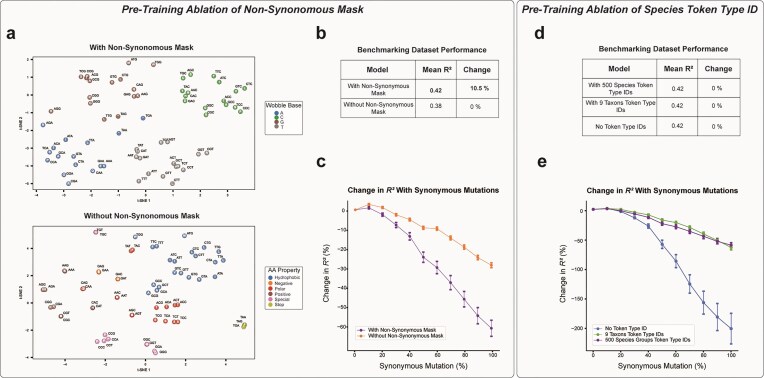
Ablation Experiments Assessing Synonym-Constrained Masking and Species Token Type IDs. (**a**) t-SNE visualization of codon embeddings for two smaller models trained with and without the synonym-constrained masking strategy. The masked model clusters by nucleotide-level features (wobble base), whereas the unmasked model clusters by amino acid identity, consistent with prior codon language models. Separation by wobble base in the model trained with the synonym masking strategy is statistically significant (PERMANOVA, pseudo-F = 5.634, *P* < .001). Meanwhile, separation by amino acid properties in the model trained without the synonym masking strategy is statistically significant (PERMANOVA, pseudo-F = 3.804, *P* < .001). (**b**) Performance comparison on benchmarking datasets: the masked model achieves a 10.5% higher mean R² across datasets and shows a statistically significant improvement (paired t-test, *P* < .05). (**c**) Sensitivity to synonymous mutations in CHO protein abundance prediction: the masked model exhibits a >60% drop in R² when sequences are mutated, while the unmasked model shows <30% drop, indicating greater reliance on codon-level context. (**d**) Benchmarking performance of models trained with 500 species-level token type IDs, 9 taxonomic-level IDs, or no token type IDs. All configurations perform similarly on standard benchmarks. (**e**) Sensitivity to synonymous mutations for the same models: the model without token type IDs shows the largest performance drop, suggesting species-specific priors help maintain robustness under codon variation.

To confirm that the unique clustering observed in Fig. 3 and the improved embedding utility shown in Fig. 4 were driven by the synonym-constrained masking strategy, we conducted controlled ablation studies. All ablation models were approximately one-tenth the size of SynCodonLM, maintaining a similar depth-to-width ratio. First, we trained a smaller model using the synonym-constrained mask and compared it to an identical model trained without masking, as implemented in prior works. Figure 5a shows that the masked model reproduced clustering by nucleotide-level features, whereas the unmasked model clustered by amino acid properties, consistent with patterns observed in existing codon language models. This validates that the distinctive clustering in SynCodonLM is attributable to the masking strategy. To ensure these results were not artifacts of t-SNE settings, we performed sensitivity analyses across multiple perplexity values (Supplementary Figs S7 and S8).

Further, we evaluated these two models on the benchmarking datasets used in Fig. 4. As shown in Fig. 5b, the model trained with the synonym-constrained mask achieved a 10.5% higher mean R² across datasets compared to the unmasked model, with a statistically significant difference (*P* < .05, paired t-test).

We next examined sensitivity to synonymous mutations in a biologically relevant task: predicting protein abundance in CHO cells. This task depends on both codon-level and protein-level information. Following the approach in CaLM [13], we introduced silent mutations at varying levels and measured performance degradation. Figure 5c shows that the masked model exhibited a >60% drop in R² when sequences were mutated, whereas the unmasked model showed <30% drop. The larger decline for the masked model indicates stronger reliance on codon-specific context rather than protein-level signals, which is desirable for tasks focused on codon optimization. Raw R² values for this experiment are provided in Supplementary Fig. S9.

### Ablation studies to evaluate usage of token type IDs to encode species specific information

To assess the impact of species-specific token type IDs, we conducted ablation studies comparing three configurations: 500 grouped-species IDs (Fig. 1c), 9 taxonomic-level IDs based on NCBI hierarchy, and no token type IDs. As shown in Fig. 5d, all configurations performed similarly on the benchmarking datasets. However, when tested under synonymous mutation (Fig. 5e), the model without token type IDs exhibited the largest performance drop, suggesting that token type IDs provide additional contextual signals that improve robustness. While these signals likely capture species-level codon usage patterns, they may also introduce indirect protein-level information, which could explain the observed resilience. Raw R² values for these experiments are provided in Supplementary Fig. S10.

To further explore this effect, we trained an additional full-size SynCodonLM model without token type IDs for scenarios requiring complete abstraction from species-level or protein-level priors. At scale, the model with token type IDs performed slightly better on benchmarks. Moreover, incorporating token type IDs enables the transformer encoder to operate in a generative fashion despite lacking a decoder block, a capability we introduced and demonstrated in our codebase upon preprint. This generative utility was later validated by another group [49], who benchmarked SynCodonLM against other codon optimization approaches and showed that our model performs competitively in practical applications.

### Necessity of full pre-training with the synonym constrained strategy

To determine whether full pre-training with our strategy was necessary, as opposed to fine-tuning a pre-existing model, we fine-tuned a model originally trained without masking using the synonym-constrained approach. Fine-tuning was applied across all layers, using ~5% of the compute required for full pre-training. Despite this, the fine-tuned model retained clustering by amino acid identity (Supplementary Fig. S11), indicating that the benefits of synonym-constrained training cannot be recovered through fine-tuning alone. This outcome is expected given weight tying between input embeddings and the language head, which likely drives fundamentally different optimization trajectories during full pre-training.

## Discussion

In this work, we introduced SynCodonLM, a codon language model trained with a synonym-constrained masked language modeling strategy. SynCodonLM is the first codon language model designed to learn nucleotide-level patterns in coding DNA, without being confounded by protein-level semantics. This distinction is evident in the learned input embeddings, which cluster codons based on nucleotide features such as wobble base and suffix dinucleotides, unlike prior models that group codons by amino acid identity.

By removing the need to infer amino acid identity during training, SynCodonLM captures codon-level variation that is both interpretable and predictive. It consistently outperforms existing codon language models on tasks where synonymous codon changes drive functional outcomes, particularly when the protein sequence is held constant.

Beyond our novel training objective, SynCodonLM benefits from two key innovations: it leverages the DeBERTa architecture for enhanced representation learning, and it is trained on over 43 million coding sequences from over 35 000 species, the largest and most diverse dataset used in codon language modeling to date.

Beyond the pre-training dataset, we aggregated several evaluation datasets specifically designed to isolate codon-level effects by varying codon usage while keeping protein sequences constant. These controlled datasets enable benchmarking of codon language models without confounding protein semantics and represent a valuable resource for the community. They can serve as standardized benchmarks for future studies and provide a clear measure of how well a model focuses on codon language independent of protein-level signals.

While these datasets provide a controlled setting for evaluating codon-level effects, they are not without limitations. Most involve model proteins such as GFP or RFP, which may not fully capture the complexity of endogenous gene regulation. Additionally, some datasets have relatively small sample sizes, which could limit statistical power. These constraints should be considered when interpreting results and highlight the need for broader benchmarks that include diverse proteins and experimental conditions.

Central to our approach is the use of logit-space constraints during training, a technique increasingly recognized in machine learning for enforcing structural priors and improving interpretability. By masking logits for non-synonymous codons, we align the model’s prediction space with biological reality, guiding it toward disentangled representations. This strategy reflects a broader principle in ML: that domain-specific constraints can reshape foundational model training to better capture latent structure. Similar approaches are emerging in symbolic reasoning, chemical modeling, and grammar-constrained generation, where output validity and semantic disentanglement are critical.

SynCodonLM has broad potential for downstream applications, especially in nucleotide-based therapeutics and biotherapeutics. In these domains, silent mutations are often used to alter biological properties. For example, codon optimization has been shown to increase vaccine efficacy [50–52], enhance viral fitness in oncolytic adenoviruses [11], increase mRNA stability and translation efficiency in AAV payloads [53, 54], improve AAV capsid packaging [55], boost therapeutic antibody yield [56–58], and influence post-translation modifications [59].

SynCodonLM could also be applied to large-scale public datasets, such as those from the Protein Abundance Database [37], to build predictive models for codon optimization. Its ability to isolate the contribution of coding sequence, independent of protein-level signals, could enable species-specific models that reveal new insights into translational regulation.

Additionally, SynCodonLM can be used in a generative fashion, despite being an encoder transformer. The utility of token type IDs for encoding species-specific codon usage patterns allows the model to design fully synthetic coding sequences, which have already been shown to be very competitive when compared to other methods for codon optimization.

Together, these capabilities position SynCodonLM not only as a powerful tool for codon-aware modeling, but also as a case study in how constraint-aware training can enhance representation learning across structured domains in biology and beyond.

## Supplementary Material

gkag166_Supplemental_File

## Data Availability

The code used for pretraining, as well as future applications of the model, is available on GitHub at https://github.com/Boehringer-Ingelheim/SynCodonLM. The trained model weights are available on Hugging Face at https://huggingface.co/jheuschkel/SynCodonLM-V2. The dataset used for pretraining the model is available on Hugging Face at https://huggingface.co/datasets/jheuschkel/clustered-cds-dataset. All datasets used for model benchmarking are available on GitHub at https://github.com/Boehringer-Ingelheim/SynCodonLM/tree/master/benchmarking-datasets. Additionally, the codebase has been stored on Zenodo at https://doi.org/10.5281/zenodo.18262433.
