# Supplementary material for: Advancing codon language modeling with synonymous codon constrained masking
Source: Nucleic Acids Res. 2026 Feb 25;54(5):gkag166. doi: 10.1093/nar/gkag166 (PMC12956333; doi:10.1093/nar/gkag166)
Supplement: gkag166_Supplemental_File [file gkag166_supplemental_file.pdf]

Supplementary Information

n	Species	Protein	Fitness Metric	Data Scaling	Citation	Name in Text
185	*E. coli*	GFP	mRNA Toxicity	Min Max	a	'mRNA Toxicity 1'
97	*E. coli*	GFP	mRNA Toxicity	Min Max	a	'mRNA Toxicity 2'
219	*E. coli*	GFP	Expression	Min Max	b	'GFP Expression'
1459	*E. coli*	mRFP	Expression	Min Max	c	'mRFP Expression'
1124	S. cerevisiae	GFP	mRNA Abundance	Yeo-Johnson	d	'GFP mRNA Abundance 1'
2432	S. cerevisiae	GFP	mRNA Abundance	Min Max	d	'GFP mRNA Abundance 2'
523	S. cerevisiae	TDH3	mRNA Abundance	Min Max	d	'TDH3 mRNA Abundance'

[a] https://doi.org/10.1073/pnas.1810022115
[b] https://doi.org/10.1186/s12864-021-07462-z
[c] https://doi.org/10.1093/nar/gkad035
[d] https://doi.org/10.1093/molbev/msx229

Supplementary Table 1: Evaluation dataset properties

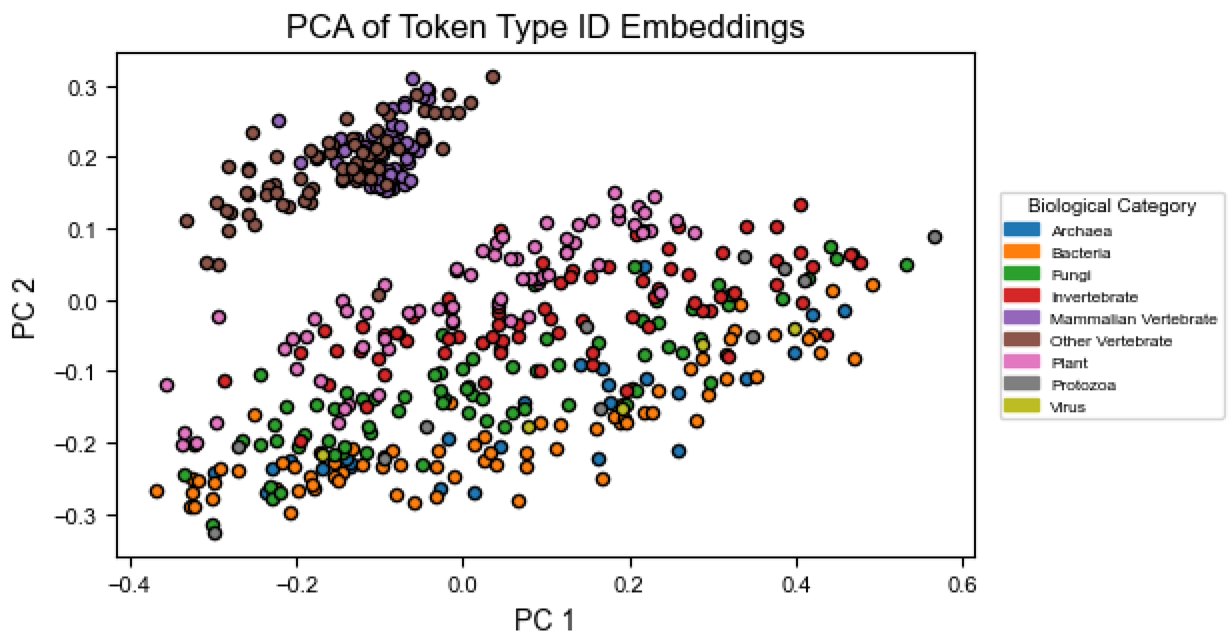

.

Supplementary Figure 1: PCA of token type ID embeddings shows grouping within biological categories. SynCodonLM appears to have learned some meaningful token type ID embeddings, as biological categories seem to be grouped near each other.

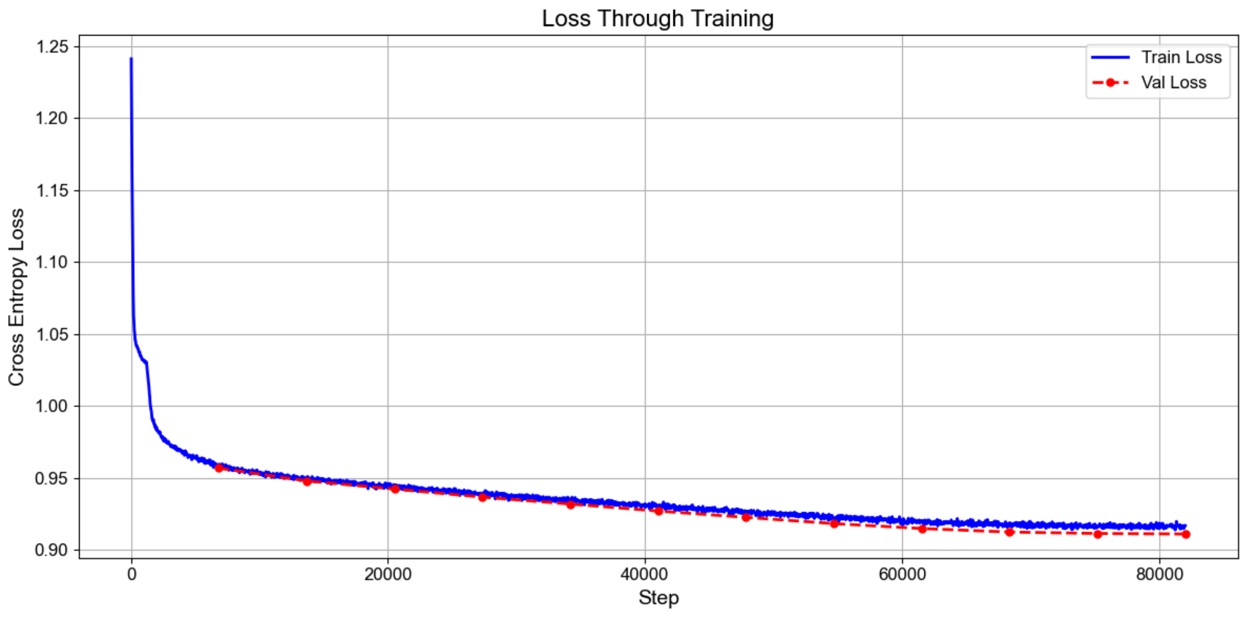

Supplementary Figure 2: Loss curve through training shows stable convergence.

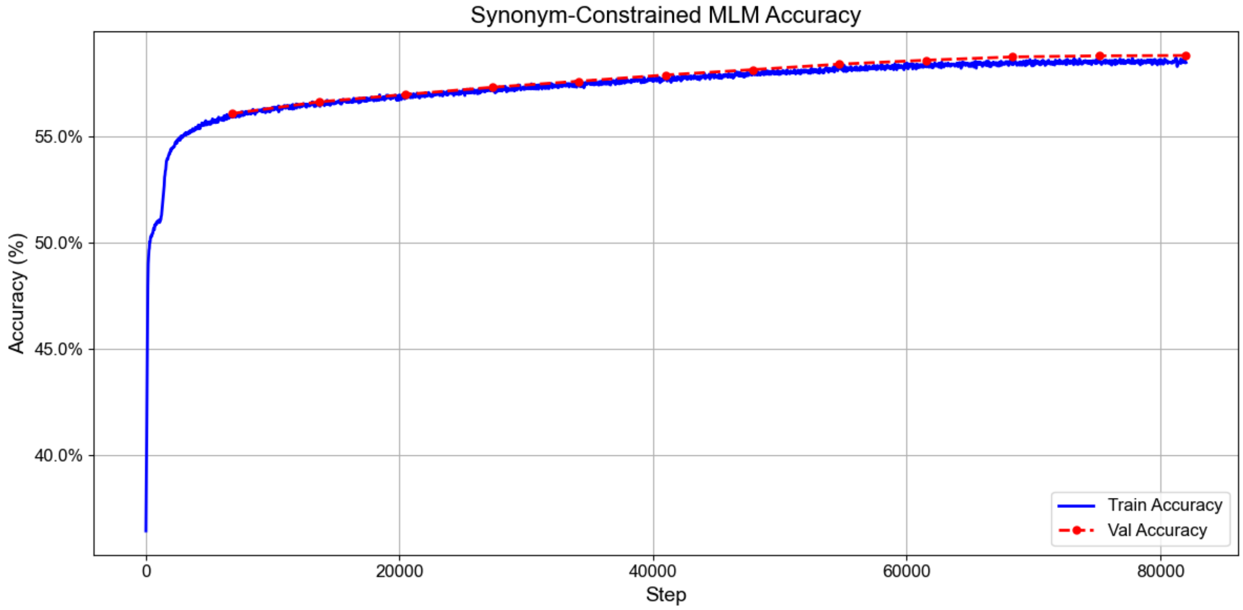

Supplementary Figure 3: Synonym-constrained masked prediction accuracy through training shows accuracy of over 60% on masked codon prediction.

a)

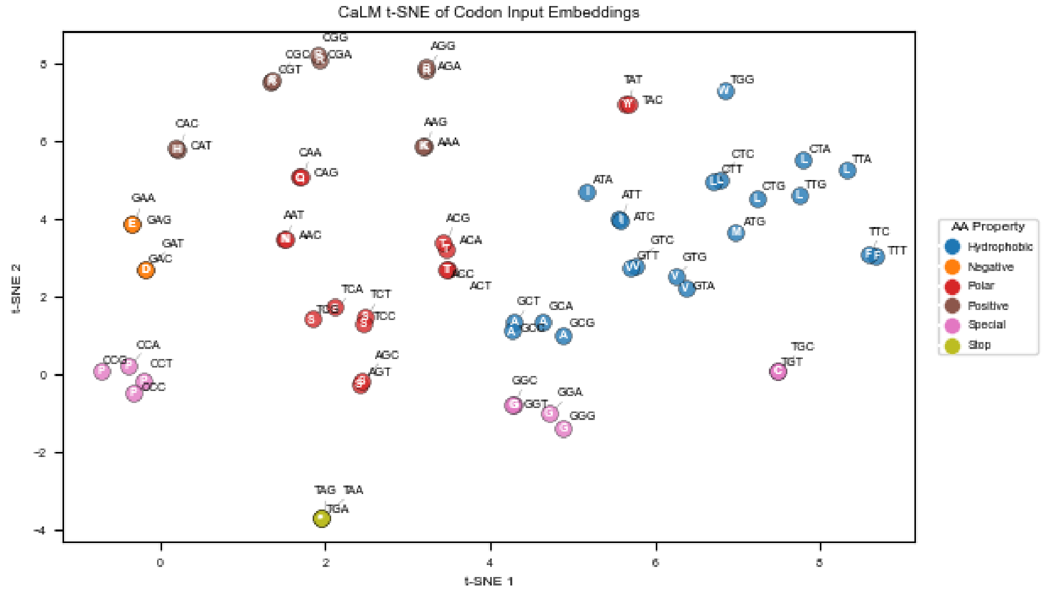
CaLM t-SNE of Codon Input Embeddings
t-SNE 1
t-SNE 2
AA Property
Hydrophobic
Negative
Polar
Positive
Special
Stop

b)

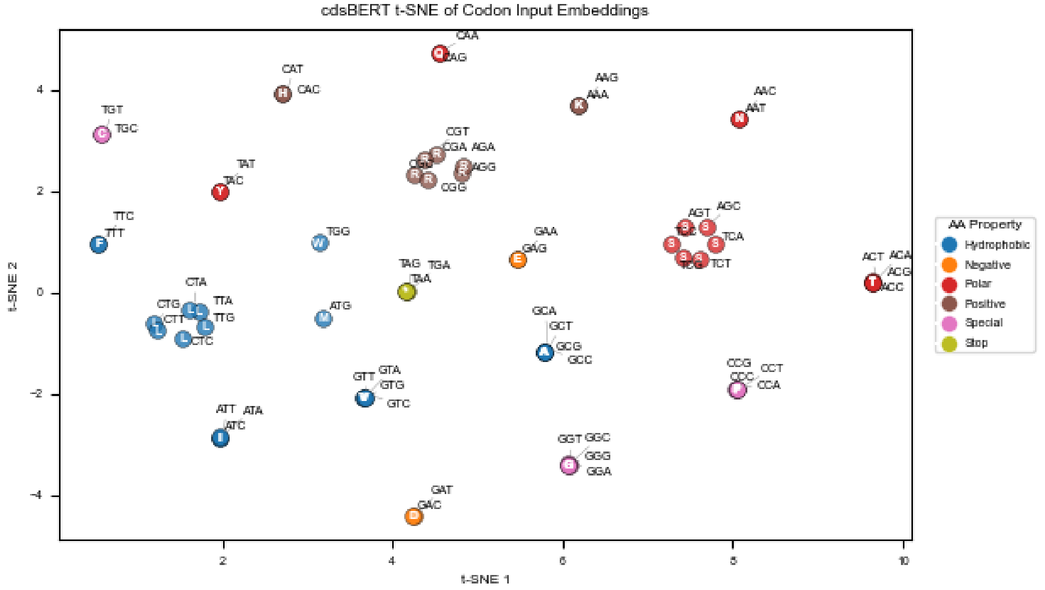
cdsBERT t-SNE of Codon Input Embeddings
t-SNE 1
t-SNE 2
AA Property
Hydrophobic
Negative
Polar
Positive
Special
Stop

c)

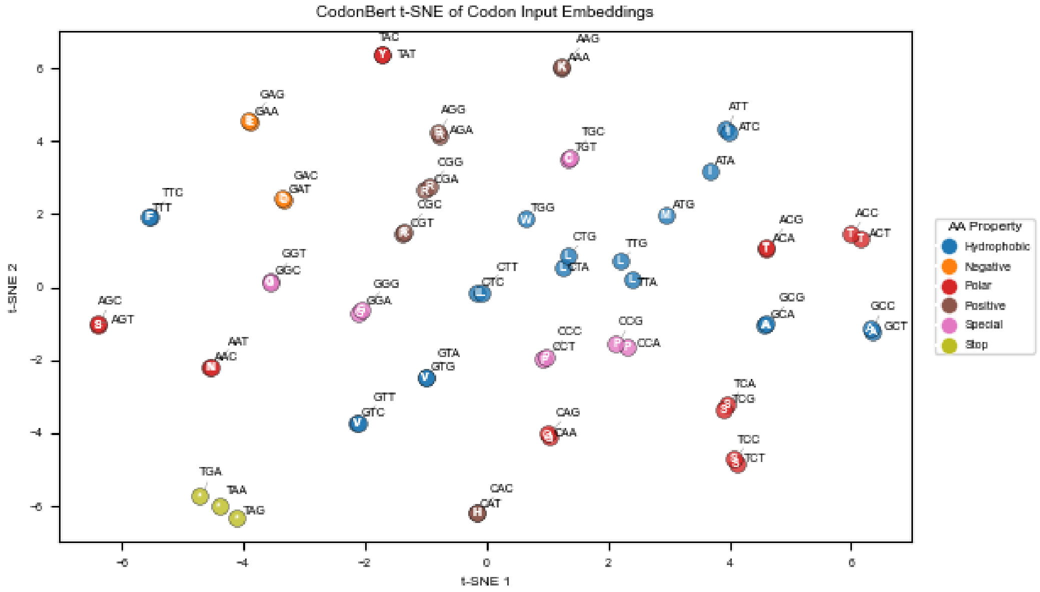
CodonBert t-SNE of Codon Input Embeddings
t-SNE 1
t-SNE 2
AA Property
Hydrophobic
Negative
Polar
Positive
Special
Stop

d)

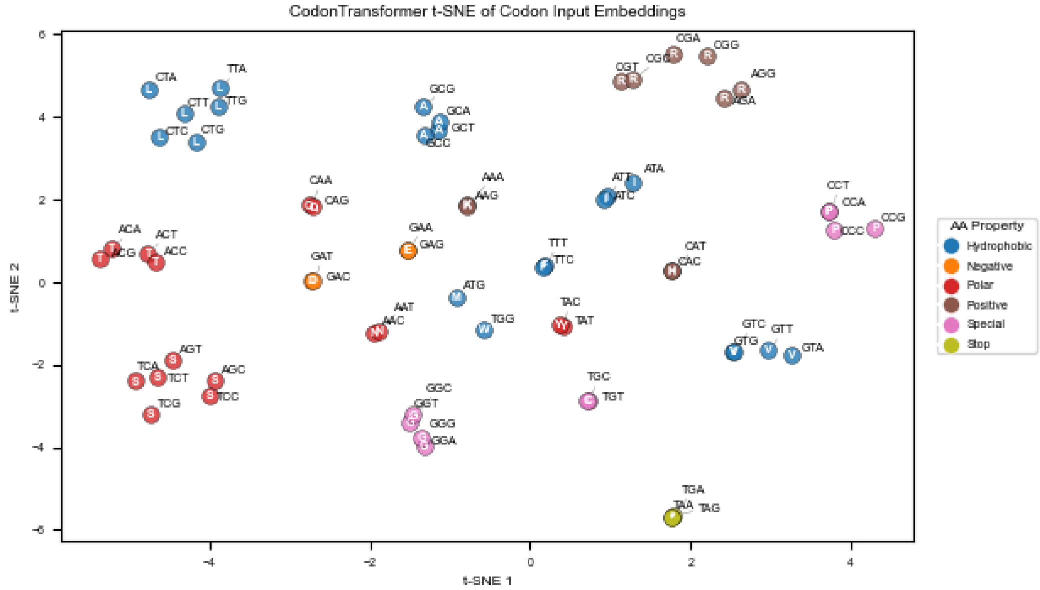
CodonTransformer t-SNE of Codon Input Embeddings
t-SNE 1
t-SNE 2
AA Property
Hydrophobic
Negative
Polar
Positive
Special
Stop

e)

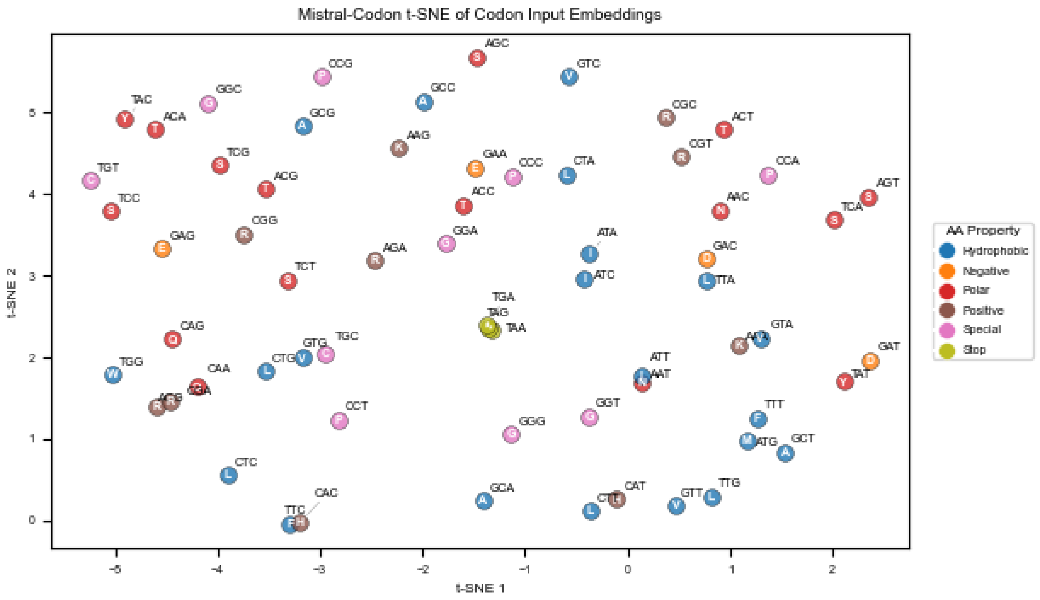

Supplementary Figure 4: t-SNE of input-codon ID embeddings using (a) CaLM, (b) cdsBERT, (c) CodonBERT, (d) CodonTransformer, and (e) Mistral Codon. In all models, codons are visibly grouped by amino acid/amino acid properties.

t-SNE Perplexities of Main Figure 3

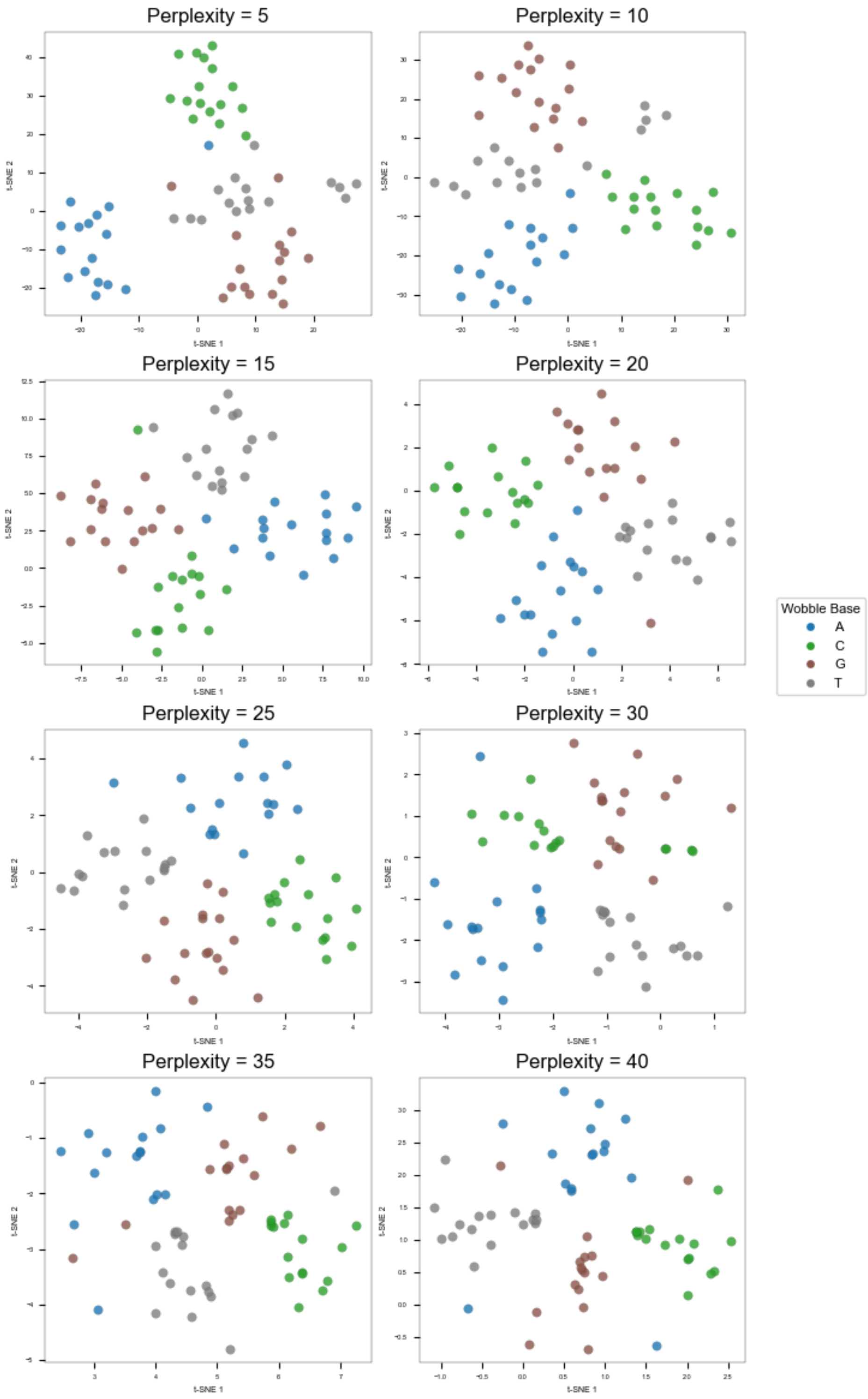

Supplementary Figure 5: t-SNE Perplexity Sensitivity Analysis of Main Figure 3.

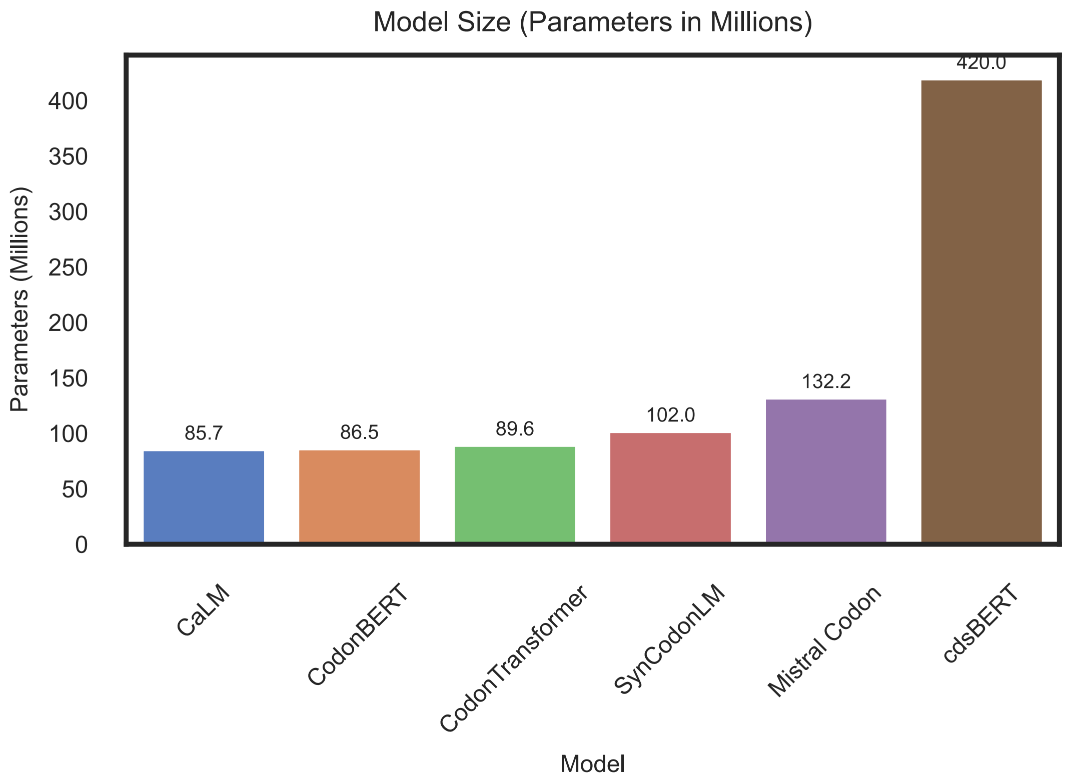

Supplementary Figure 6: Model size comparison.

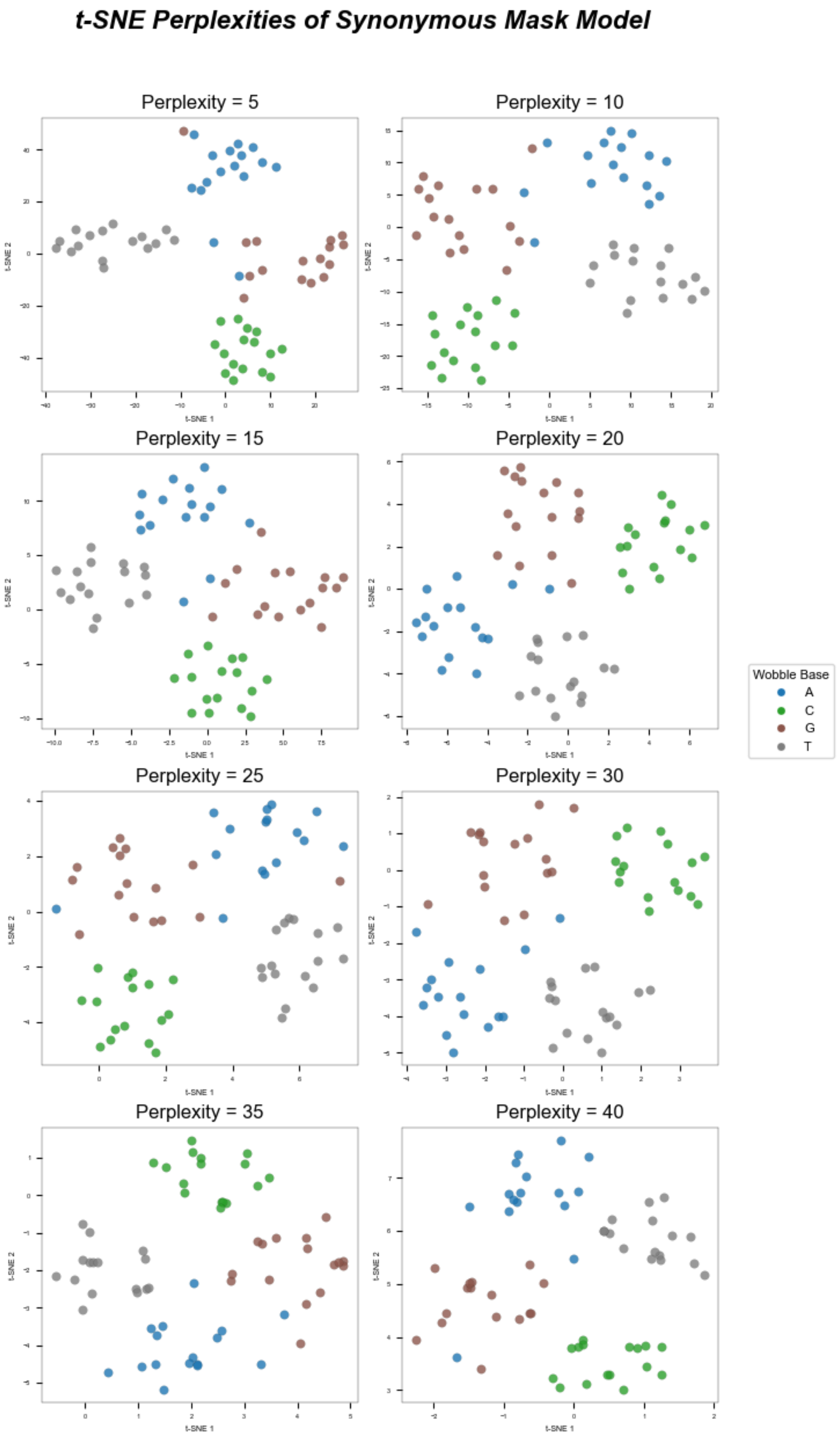

Supplementary Figure 7: t-SNE Perplexity Sensitivity Analysis of Main Figure 5a Model Trained With Synonymous Masking Strategy.

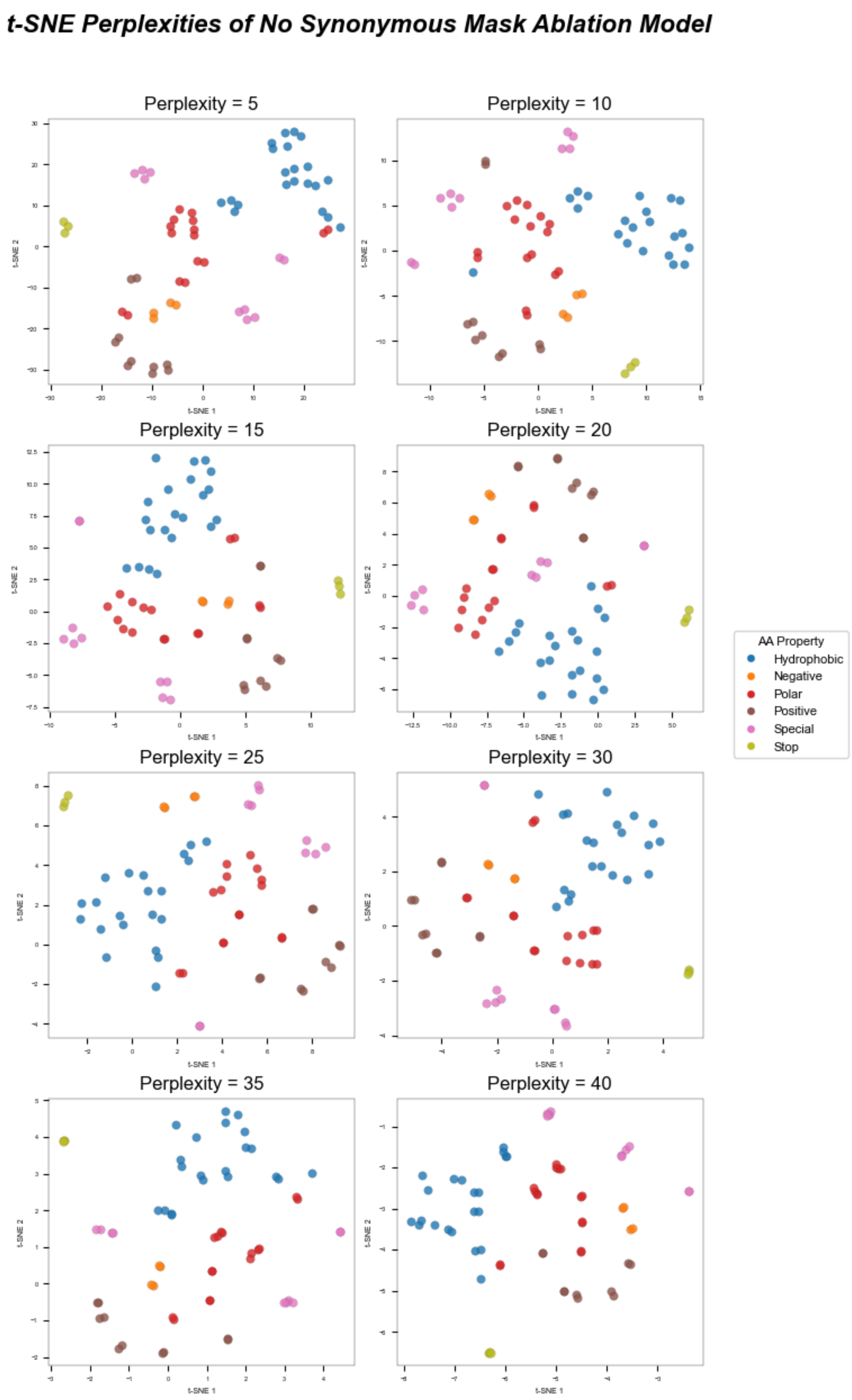

Supplementary Figure 8: t-SNE Perplexity Sensitivity Analysis of Main Figure 5a, Model Trained Without Synonymous Masking Strategy.

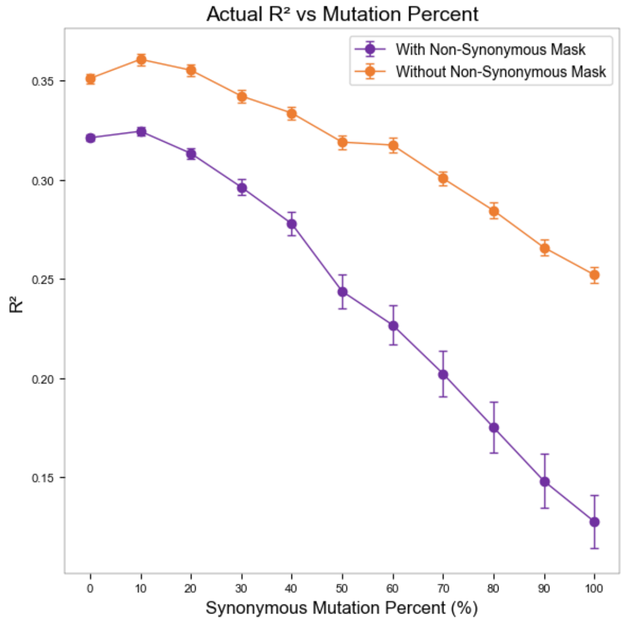

Supplementary Figure 9: Actual R^2 Values from Main Figure 5c.

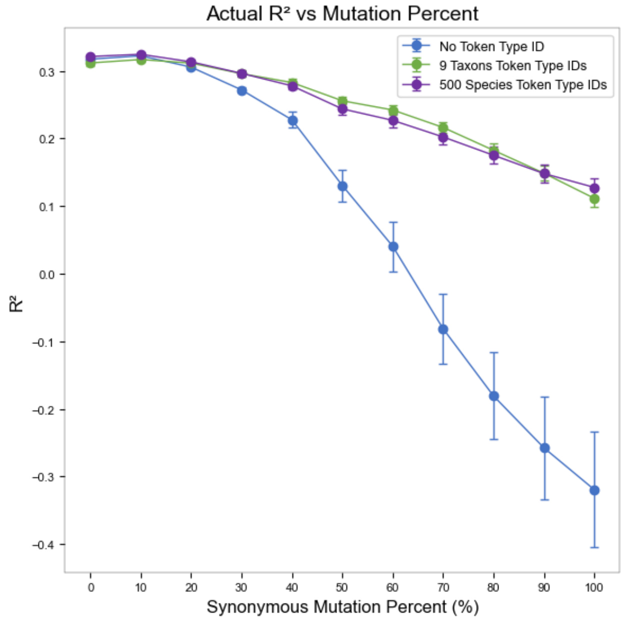

Supplementary Figure 10: Actual R^2 Values from Main Figure 5e.

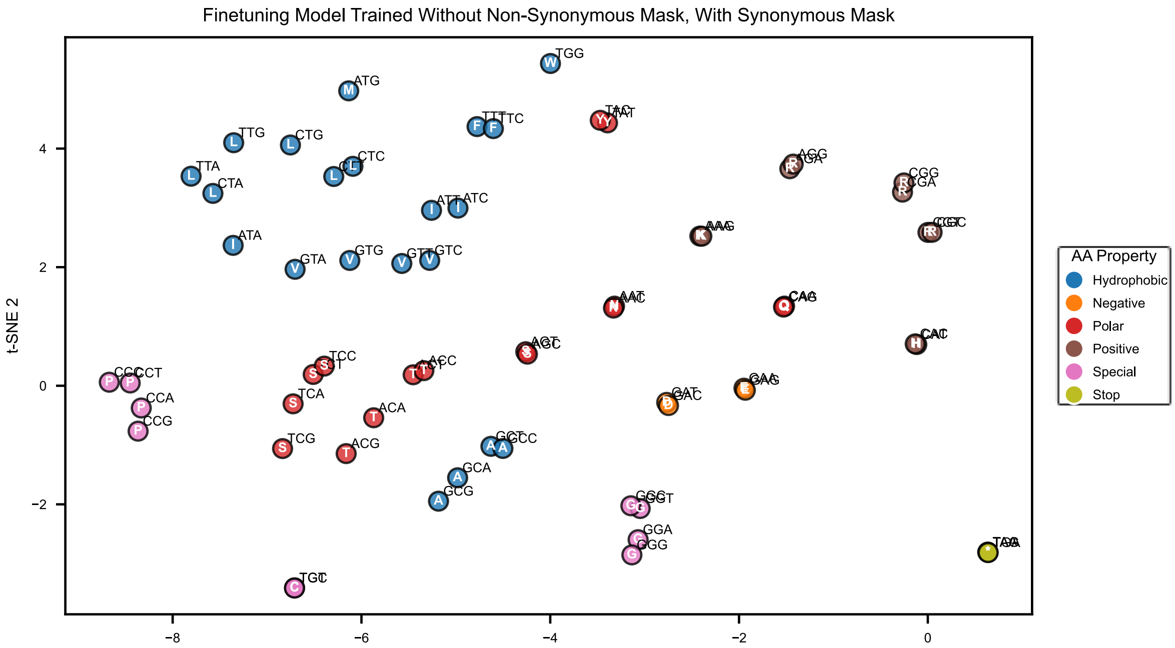

Supplementary Figure 11: Finetuning our Ablation Model Trained Without a Non-Synonymous Mask, With a Synonymous Mask. Notably, input embeddings have little change to the way they cluster (by AA property), indicating that the synonymous mask is most useful when fully pretraining a codon language model.
